# Type 2 Diabetes and Its Association With Psychiatric Disorders in Young Adults in South Korea

**DOI:** 10.1001/jamanetworkopen.2023.19132

**Published:** 2023-06-30

**Authors:** Min-Kyung Lee, Su-Young Lee, Seo-Young Sohn, Jiyeon Ahn, Kyungdo Han, Jae-Hyuk Lee

**Affiliations:** 1 Division of Endocrinology and Metabolism, Department of Internal Medicine, Myongji Hospital, Hanyang University Medical Center, Gyeonggi-do, Republic of Korea; 2 Department of Psychiatry, Myongji Hospital, Hanyang University Medical Center, Gyeonggi-do, Republic of Korea; 3 Department of Statistics and Actuarial Science, Soongsil University, Seoul, Republic of Korea

## Abstract

**Importance:**

Because type 2 diabetes (T2D) has become increasingly prevalent among young adults, the study of the association of T2D with psychiatric disorders in young adults is important for early detection and timely intervention.

**Objective:**

To determine whether a diagnosis of a psychiatric disorder is associated with increased risk of developing T2D in young adults.

**Design, Setting, and Participants:**

This large-scale prospective cohort study used data collected by the South Korean National Health Insurance Service between 2009 and 2012, representing 97% of the South Korean population. Young adults aged 20 to 39 years with and without diagnoses of psychiatric disorders were included in the study. Young adults with missing data and those with a history of T2D were excluded from the study. The cohort was followed up to monitor development of T2D until December 2018. Data were analyzed from March 2021 to February 2022.

**Exposure:**

Diagnosis of 1 of 5 psychiatric disorders, including schizophrenia, bipolar disorder, depressive disorder, anxiety disorder, and sleep disorder.

**Main Outcomes and Measures:**

The primary outcome was newly diagnosed T2D during a follow-up period of 7.59 years. The incidence rate of T2D was calculated as the number of new cases per 1000 person-years during the follow-up period. The Cox proportional hazards regression model was used to estimate the hazard ratios (HRs) and 95% CIs for T2D incidence. Exploratory analyses were performed for subgroups stratified by age and sex.

**Results:**

In total, 6 457 991 young adults (mean [SD] age, 30.74 [4.98] years; 3 821 858 men [59.18%]) were followed up, including 658 430 individuals with psychiatric disorders. The cumulative incidence of T2D differed significantly between individuals with and without psychiatric disorders (log-rank test, *P* < .001). Incidence rates of T2D for individuals with and without psychiatric disorders were 2.89 and 2.56 per 1000 person-years, respectively. Individuals with a diagnosis of any psychiatric disorder showed a higher risk of developing T2D than those without a diagnosis (adjusted HR, 1.20; 95% CI, 1.17-1.22). The adjusted HRs for T2D were 2.04 (95% CI, 1.83-2.28) for individuals with schizophrenia, 1.91 (95% CI, 1.73-2.12) for individuals with bipolar disorder, 1.24 (95% CI, 1.20-1.28) for individuals with depressive disorder, 1.13 (95% CI, 1.11-1.16) for individuals with anxiety disorder, and 1.31 (95% CI, 1.27-1.35) for individuals with sleep disorder.

**Conclusions and Relevance:**

In this large-scale prospective cohort study of young adults, 5 psychiatric disorders were significantly associated with an increased risk of developing T2D. Young adults with schizophrenia and bipolar disorder in particular were at a higher risk of T2D. These results have important implications for early detection of and timely intervention in T2D for young adults with psychiatric disorders.

## Key Points

**Question:**

Do young adults with psychiatric disorders have an increased risk of developing type 2 diabetes?

**Findings:**

In this cohort study of more than 6.4 million young adults in South Korea, a diagnosis of a psychiatric disorder, particularly schizophrenia and bipolar disorder, was significantly associated with an increased risk of developing type 2 diabetes.

**Meaning:**

These results have important implications for early detection and timely intervention of type 2 diabetes in young adults with psychiatric disorders.

## Teaser

This cohort study assesses the association of various psychiatric disorders with the incidence of type 2 diabetes in young adults in South Korea.

## Introduction

The prevalence of type 2 diabetes (T2D) in young adults is increasing worldwide.^1^ Young-onset T2D (defined as young adults aged <40 years) rapidly progresses, resulting in early and frequent development of microvascular and macrovascular complications and premature death, mainly due to longer disease exposure.^2,3^ T2D has unfavorable long-term outcomes and can be a serious public health problem.^4^ Similar to late-onset T2D, the main factors associated with an increased risk of young-onset T2D are obesity, physical activity, sedentary lifestyle, and socioeconomic status.^5,6^ Young-onset T2D is associated with excessive morbidity and an unexpectedly large incidence of psychiatric disorders.^7^

Psychiatric disorders are among the most prevalent health problems affecting the general population worldwide.^8^ Common psychiatric disorders, including depressive disorder, bipolar disorder, and anxiety disorder, are more prevalent among young adults in their 20s than those in their 30s or 40s.^9^ Psychiatric disorders are associated with a higher risk of subsequent medical conditions,^10^ and the prevalence of T2D ranges in patients with psychiatric disorders from 5% to 22%, depending on the psychiatric disorder.^11^ In a 2022 population-based cohort study, Lindekilde et al^12^ reported that the incidence of T2D increased across different categories of psychiatric disorders and was particularly high in younger people. Another study^13^ indicated that diagnosis of a psychiatric disorder was associated with an earlier onset of diabetes-related complications and death. The global burden of psychiatric disorders continues to grow,^14^ and recognizing these conditions early may be beneficial to prevent T2D-related morbidity and mortality. Thus, a more detailed study focused on a broad range of psychiatric disorders and T2D in young adults is necessary.

This large-scale, prospective, cohort study aimed to examine whether common psychiatric disorders (schizophrenia, bipolar disorder, depressive disorder, anxiety disorder, and sleep disorder) are associated with a higher risk of developing T2D in young adults. Additionally, we aimed to investigate the age-specific and sex-specific factors associated with developing T2D in young adults with psychiatric disorders.

## Methods

This study was approved by the institutional review board of Myongji Hospital and conforms to the ethical guidelines of the World Medical Association Declaration of Helsinki.^15^ The requirement for informed consent was waived because the data were publicly anonymized under confidentiality guidelines. The study design and analysis followed the Strengthening the Reporting of Observational Studies in Epidemiology (STROBE) reporting guideline.

### Data Sources and Study Population

We designed a nationwide population-based cohort study and obtained information from the South Korean National Health Insurance Service (NHIS), which represents 97% of the South Korean population. The NHIS data uses a stratified random sampling method based on age, sex, and participants’ eligibility status and conducts biennial health examinations for all Korean employees or citizens older than 40 years.^16^ The database contains sociodemographic data, self-reported questionnaires on lifestyle behaviors, anthropometric measurements, laboratory test results, medical diagnoses based on the *International Statistical Classification of Diseases, Tenth Revision, Clinical Modification (ICD-10-CM)*, and treatment data of the Korean population.

This study initially included 6 891 399 participants aged 20 to 39 years who underwent a national general health examination between 2009 and 2012. Participants with missing data (287 346 individuals) and those with a history of diabetes (139 797 individuals) were excluded (eFigure in Supplement 1). Additionally, we excluded 6205 participants who developed T2D within the first year of the study period. Participants were followed up for T2D incidence from 1 year after the day of the health examination until December 31, 2018.

###  Psychiatric Disorders

Psychiatric disorders at diagnosis were defined according to subcategories from *ICD-10-CM* and the Diagnostic Research Criteria subchapter F.^17,18^ We included 5 categories of common psychiatric disorders: schizophrenia (*ICD-10-CM* code F20), bipolar disorder (*ICD-10-CM* codes F30-F31), depressive disorder (*ICD-10-CM* codes F32-F33), anxiety disorder (*ICD-10-CM* codes F40-F41), and sleep disorder (*ICD-10-CM* codes G47 and F51). We selected major psychiatric disorders, which have relatively high prevalence in young adults. Alcohol use disorder and eating disorder were not included, but we considered alcohol consumption as a covariate in the analysis. The date of diagnosis for each psychiatric disorder was defined as the earliest date of a service claim with *ICD-10-CM* codes for psychiatric disorders within 5 years before the 2009 health examination.

### Study Outcomes: T2D and Follow-Up

In this study, the primary outcome was newly diagnosed T2D, and we used the operational definition of T2D.^19^ Participants were classified as having T2D when they had at least 1 service claim of *ICD-10-CM* codes E11, E12, E13, or E14, and received a prescription for at least 1 antidiabetic drug. Using the NHIS national health examination database, participants with fasting plasma glucose levels greater than or equal to 126 mg/dL (to convert to millimoles per liter, multiply by 0.0555) without a claim for antidiabetic medication under the *ICD-10-CM* code were also included. Participants with type 1 diabetes, gestational diabetes, or missing data were excluded.

The study participants were followed up from the time of study entry until the date of T2D diagnosis, emigration, death, or end of the study period (whichever came first). For each psychiatric disorder, we estimated the T2D incidence during the follow-up period in individuals with and without psychiatric disorders. Moreover, we analyzed subgroups according to age group (20-29 years and 30-39 years) and sex (male and female).

### Measurements and Definition of Covariates

The following variables were included as covariates for their association with psychiatric disorders and T2D in the multivariate analyses: age, sex, annual income, alcohol consumption, cigarette smoking, and physical activity level. Heavy drinking was defined as weekly alcohol consumption of more than 28 standard drinks (210 g of alcohol) with a calculated alcohol content of 7.5 g of alcohol in 1 standard drink. Regular physical activity was defined as performing more than 30 minutes of moderate-intensity activity at least 5 times a week or more than 20 minutes of vigorous-intensity activity at least 3 times a week. Individuals who paid the bottom 20% of health insurance premiums were considered as the low-income group. Height, weight, and blood pressure were measured during regular medical examinations according to standardized methods. Body mass index was calculated as weight in kilograms divided by height in meters squared. Blood samples were obtained to measure fasting plasma glucose and total cholesterol levels after overnight fasting before each examination. The hospitals where these health examinations were performed were certified by the NHIS and subjected to regular quality control. Comorbidities were defined using a combination of *ICD-10-CM* codes and self-reported medication histories. The presence of hypertension was defined according to *ICD-10-CM* codes I10 to I13 or I15, prescription of an antihypertensive agent, or systolic and diastolic blood pressure greater than or equal to 140/90 mm Hg. Dyslipidemia was defined as at least 1 claim per year for the prescription of an antidyslipidemic agent under the *ICD-10-CM* code E78. Metabolic syndrome was defined as a combination of abdominal obesity, impaired fasting glucose, atherogenic dyslipidemia, and elevated blood pressure according to the revised National Cholesterol Education Program Adult Treatment Panel III criteria.^20^

### Statistical Analysis

Baseline characteristics were compared using *t* tests and χ^2^ tests for continuous and categorical variables, respectively. Data were presented as mean (SD) or proportions (percentages). The incidence rate of T2D was calculated as the number of new cases per 1000 person-years during the follow-up period. The disease-free probability of T2D was calculated using Kaplan-Meier curves, and a log-rank test was performed to analyze differences between the groups. The Cox proportional hazards regression model was used to estimate the hazard ratios (HRs) and 95% CIs for T2D incidence. We performed multivariate adjustments for confounding factors. Model 1 was unadjusted; model 2 was adjusted for age, sex, income, alcohol consumption, smoking status, and physical activity; and model 3 was adjusted for age, sex, income, alcohol consumption, smoking status, physical activity, and metabolic syndrome at baseline. We further performed exploratory analyses for subgroups stratified by age and sex, including interaction terms between psychiatric disorders and subgroups. We also performed a propensity score matching analysis using the 1:5 nearest neighbor matching method (caliper width 0.2 of the SD of the logit propensity score) to test the robustness of the original results. Statistical significance was defined as a 2-sided *P* < .05. All analyses were performed using SAS statistical software version 9.4 (SAS Institute). We performed data analysis from March 2021 to February 2022.

## Results

### General Baseline Characteristics

The final cohort included 6 457 991 participants (3 821 858 men [59.18%]; mean [SD] age, 30.74 [4.98] years). The baseline characteristics of the study population, including participants with and without psychiatric disorders, are presented in Table 1. Of the participants, 5 799 561 (89.81%) had no diagnosis of a psychiatric disorder and 658 430 (10.19%) had a diagnosis of a psychiatric disorder. Among participants with psychiatric disorders, 7408 individuals (1.13%) had schizophrenia, 10 811 (1.64%) had bipolar disorder, 181 230 (27.52%) had depressive disorder, 408 415 (62.03%) had anxiety disorder, and 170 039 (25.82%) had sleep disorder. At baseline, the percentage of low-income patients was 15.75% (1 017 072 patients). The proportion of women with psychiatric disorders was higher than the proportion of women without psychiatric disorders (357 913 patients [54.36%] vs 2 278 220 patients [39.28%]). eTables 1 to 5 in Supplement 1 present the descriptive statistics of participants with and without different psychiatric disorders.

**Table 1.** Baseline Characteristics of the Young Adult Cohort With and Without Diagnosis of a Psychiatric Disorder

Characteristic	Participants, No. (%)		
	Total (N = 6 457 991)	Without diagnosis of a psychiatric disorder (n = 5 799 561)	With diagnosis of psychiatric disorder (n = 658 430)^a^
Psychiatric disorder type			
Schizophrenia	7408 (0.11)	NA	7408 (1.13)
Bipolar disorder	10 811 (0.17)	NA	10 811 (1.64)
Depressive disorder	181 230 (2.81)	NA	181 230 (27.52)
Anxiety disorder	408 415 (6.32)	NA	408 415 (62.03)
Sleep disorder	170 039 (2.63)	NA	170 039 (25.82)
Age, mean (SD), y	30.80 (4.98)	30.74 (4.98)	31.36 (4.99)
Sex			
Male	3 821 858 (59.18)	3 521 341 (60.72)	300 517 (45.64)
Female	2 636 133 (40.82)	2 278 220 (39.28)	357 913 (54.36)
Health factors			
Low income	1 017 072 (15.75)	897 429 (15.47)	119 643 (18.17)
Heavy alcohol drinker	562 333 (8.71)	512 236 (8.83)	50 097 (7.61)
Current smoker	2 234 146 (34.6)	2 048 812 (35.33)	185 334 (28.15)
Regular physical activity	830 779 (12.86)	744 991 (12.85)	85 788 (13.03)
Body mass index, mean (SD)^b^	22.95 (3.57)	22.99 (3.56)	22.61 (3.59)
Systolic blood pressure, mean (SD), mm Hg	117.58 (13.10)	117.76 (13.10)	115.96 (13.00)
Diastolic blood pressure, mean (SD), mm Hg	73.68 (9.40)	73.78 (9.40)	72.79 (9.35)
Fasting plasma glucose, mean (SD), mg/dL	89.49 (10.61)	89.52 (10.62)	89.2 (10.57)
Total cholesterol, mean (SD), mg/dL	184.42 (35.92)	184.51 (35.94)	183.66 (35.74)
Comorbidities			
Hypertension	453 867 (7.03)	408 830 (7.05)	45 037 (6.84)
Dyslipidemia	423 581 (6.56)	378 765 (6.53)	44 816 (6.81)
Metabolic syndrome	637 193 (9.87)	576 555 (9.94)	60 638 (9.21)
Follow-up duration, mean (SD), y	7.59 (6.47-8.23)	7.61 (6.49-8.24)	7.48 (6.30-8.20)

SI conversion factors: To convert cholesterol to millimoles per liter, multiply by 0.0259; glucose to millimoles per liter, multiply by 0.0555.

^a^ Totals in this column may add up to be more than 658 430 due to some participants having more than 1 diagnosis.

^b^ Body mass index is defined as calculated as weight in kilograms divided by height in meters squared.

### T2D Risk by Psychiatric Disorder

During the follow-up period of 7.59 years, 122 603 young adults with newly diagnosed T2D were identified. Of the 658 430 individuals with psychiatric disorders, 13 739 individuals (2.09%) developed T2D. The Figure presents the cumulative incidence of T2D for individuals with a specific psychiatric disorder and without such a disorder. Overall, the incidence of T2D was significantly higher in individuals with a psychiatric disorder than in those without such a disorder (log-rank test, *P* < .001). In particular, the differences were high between the patients with and without schizophrenia and between the patients with and without bipolar disorder.

**Figure.**
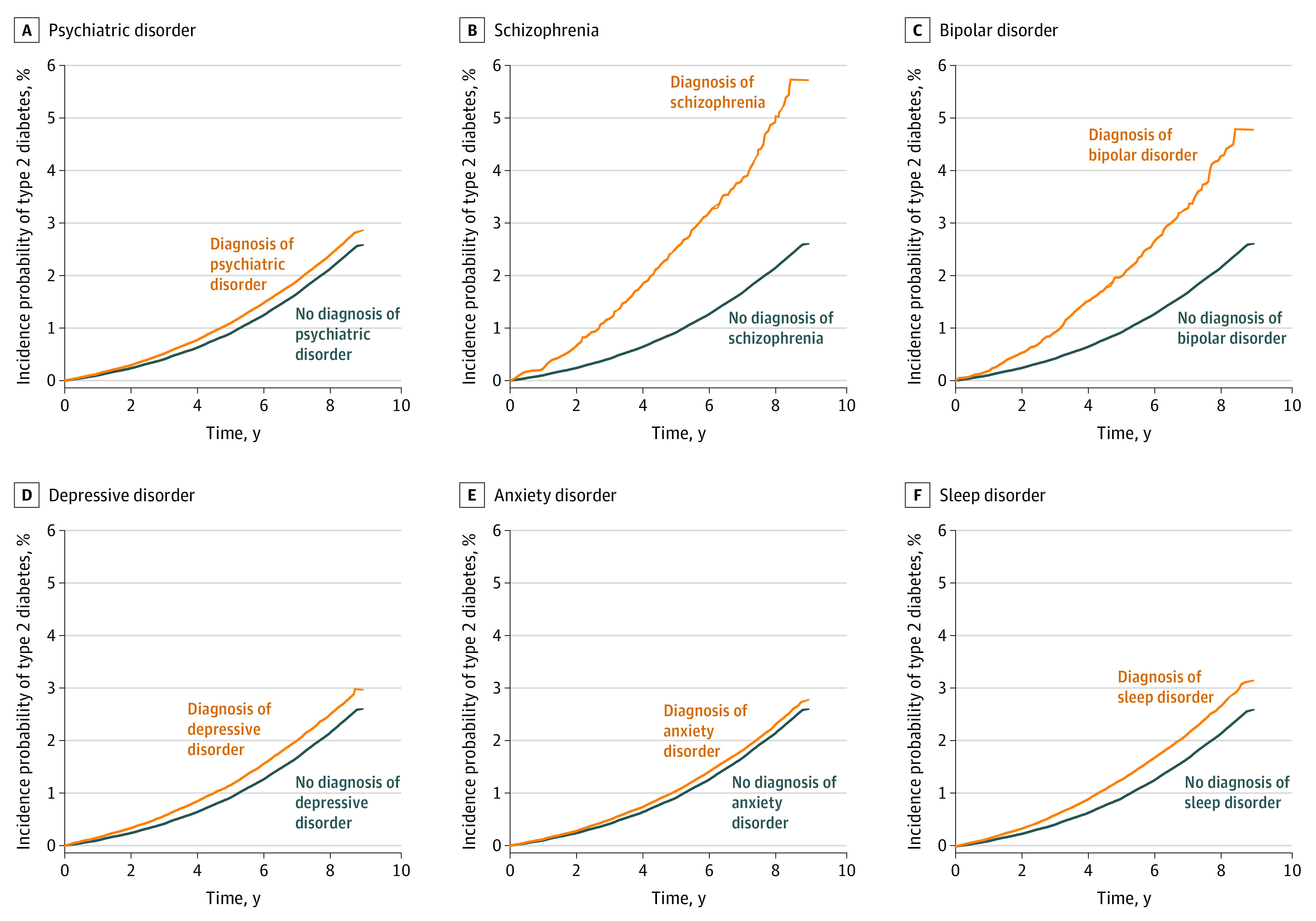
Cumulative Incidence of Type 2 Diabetes by 5 Categories of Psychiatric Disorders Graphs show the incidence rate of type 2 diabetes for all patients in the cohort with and without diagnosed psychiatric disorders (A), patients with schizophrenia (B), patients with bipolar disorder (C), patients with depressive disorder (D), patients with anxiety disorder (E), and patients with sleep disorder (F).

Table 2 presents the incidence rates and adjusted HRs (aHRs) of T2D in individuals with and without psychiatric disorders. The incidence rate of T2D for those with a psychiatric disorder was 2.89 cases per 1000 person-years, whereas the incidence rate for individuals without a psychiatric disorder was 2.56 cases per 1000 person-years. The incidence rate of T2D (per 1000 person-years) was 6.05 for individuals with schizophrenia, 5.02 for individuals with bipolar disorder, 3.00 for individuals with depression, 2.78 for individuals with anxiety disorder, and 3.23 for individuals with sleep disorder. In model 3, the multivariate aHRs for T2D were 1.20 (95% CI, 1.17-1.22) for any psychiatric disorder 2.04 (95% CI, 1.83-2.28) for individuals with schizophrenia, 1.91 (95% CI, 1.73-2.12) for individuals with bipolar disorder, 1.24 (95% CI, 1.20-1.28) for individuals with depressive disorder, 1.13 (95% CI, 1.11-1.16) for individuals with anxiety disorder, and 1.31 (95% CI, 1.27-1.35) for individuals with sleep disorder.

**Table 2.** Multivariate aHRs for Type 2 Diabetes by Psychiatric Disorder

Psychiatric disorder diagnosis	Patients, No.	Diagnosis of type 2 diabetes, No.	Follow-up duration, person-years	Incidence rate per 1000 person-years	aHR (95%CI)^a^		
					Model 1	Model 2	Model 3
Any psychiatric disorder							
No diagnosis	5 799 561	108 864	42 454 193.60	2.56	1 [Reference]	1 [Reference]	1 [Reference]
Diagnosis	658 430	13 739	4 755 135.78	2.89	1.14 (1.12-1.16)	1.22 (1.19-1.24)	1.20 (1.17-1.22)
Schizophrenia							
No diagnosis	6 450 583	122 290	47 157 606.26	2.59	1 [Reference]	1 [Reference]	1 [Reference]
Diagnosis	7408	313	51 723.13	6.05	2.405 (2.154-2.687)	2.34 (2.10-2.61)	2.040 (1.83-2.28)
Bipolar disorder							
No diagnosis	6 447 180	122 229	47 134 841.28	2.59	1 [Reference]	1 [Reference]	1 [Reference]
Diagnosis	10 811	374	74 488.10	5.02	2.01 (1.82-2.23)	2.14 (1.93-2.37)	1.91 (1.73-2.12)
Depressive disorder							
No diagnosis	6 276 761	118 701	45 909 773.02	2.59	1 [Reference]	1 [Reference]	1 [Reference]
Diagnosis	181 230	3902	1 299 556.36	3.00	1.18 (1.14-1.22)	1.26 (1.22-1.30)	1.24 (1.20-1.28)
Anxiety disorder							
No diagnosis	6 049 576	114 398	44 252 472.86	2.59	1 [Reference]	1 [Reference]	1 [Reference]
Diagnosis	408 415	8205	2 956 856.52	2.78	1.08 (1.06-1.11)	1.15 (1.12-1.17)	1.13 (1.11-1.16)
Sleep disorder							
No diagnosis	6 287 952	118 654	45 988 327.70	2.58	1 [Reference]	1 [Reference]	1 [Reference]
Diagnosis	170 039	3949	1 221 001.68	3.23	1.27 (1.23-1.31)	1.35 (1.31-1.39)	1.31 (1.27-1.35)

Abbreviation: aHR, adjusted hazard ratio.

^a^ Model 1 was crude. Model 2 was adjusted for age, sex, income, alcohol consumption, smoking status, and physical activity. Model 3 was adjusted for age, sex, income, alcohol consumption, smoking status, physical activity, and metabolic syndrome.

### T2D Risk by Psychiatric Disorders Stratified by Age, Sex, and Income

Table 3 presents aHRs for T2D incidence according to the prevalence of psychiatric disorders in subgroups stratified by age (20-29 years and 30-39 years) and sex. In the subgroup analyses, a significant interaction with psychiatric disorders was observed for both age and sex (*P* for interaction, <.001); the multivariate aHRs for T2D were higher for individuals in the 20 to 29 year age group (aHR, 1.29; 95% CI, 1.24-1.33) than in the 30 to 39 year age group (aHR, 1.17; 95% CI, 1.15-1.20), and were higher in women (aHR, 1.29; 95% CI, 1.25-1.33) than in men (aHR, 1.15; 95% CI, 1.13-1.17). Among individuals with a specific psychiatric disorder, HRs for T2D by sex indicated the same results as those of any psychiatric disorder. The difference in HR for T2D by age was observed among individuals with depressive disorder, anxiety disorder, and sleep disorder; there were no age-related differences among individuals with schizophrenia (*P* for interaction, .55) and bipolar disorder (*P* for interaction, .14).

**Table 3.** Age-Specific and Sex-Specific aHRs of Association of Type 2 Diabetes With Psychiatric Disorders

Psychiatric Disorder	aHR (95% CI)^a^	*P* value for interaction
Any		
Age, y		
20-29	1.29 (1.24-1.33)	<.001
30-39	1.17 (1.15-1.20)	
Sex		
Male	1.15 (1.13-1.17)	<.001
Female	1.29 (1.25-1.33)	
Schizophrenia		
Age, y		
20-29	2.10 (1.65-2.67)	.55
30-39	2.03 (1.79-2.30)	
Sex		
Male	1.73 (1.50-1.99)	<.001
Female	2.67 (2.22-3.20)	
Bipolar disorder		
Age, y		
20-29	2.15 (1.74-2.65)	.14
30-39	1.86 (1.65-2.08)	
Sex		
Male	1.6 (1.40-1.83)	<.001
Female	2.366 (2.03-2.76)	
Depressive disorder		
Age, y		
20-29	1.42 (1.32-1.51)	<.001
30-39	1.20 (1.16-1.24)	
Sex		
Male	1.19 (1.14-1.23)	<.001
Female	1.33 (1.26-1.40)	
Anxiety disorder		
Age, y		
20-29	1.19 (1.13-1.24)	.02
30-39	1.12 (1.09-1.15)	
Sex		
Male	1.10 (1.07-1.13)	<.001
Female	1.20 (1.15-1.25)	
Sleep disorder		
Age, y		
20-29	1.43 (1.34-1.54)	<.001
30-39	1.28 (1.23-1.32)	
Sex		
Male	1.25 (1.20-1.3)	<.001
Female	1.39 (1.32-1.47)	

Abbreviation: aHR, adjusted hazard ratio.

^a^ Adjusted for age, sex, income, alcohol consumption, smoking status, physical activity, and metabolic syndrome.

### T2D Risk by Psychiatric Disorders Adjusted by Propensity Score Matching

We further analyzed the association of T2D with psychiatric disorders using propensity score matching (eTable 6 in Supplement 1). The HRs for T2D were 1.04 (95% CI, 1.02-1.06) for individuals with any psychiatric disorder, 2.20 (95% CI, 1.97-2.46) for individuals with schizophrenia, 1.84 (95% CI, 1.67-2.04) for individuals with bipolar disorder, 1.08 (95% CI, 1.05-1.12) for individuals with depression, 0.99 (95% CI, 0.97-1.01) for individuals with anxiety disorder, and 1.17 (95% CI, 1.13-1.20) for individuals with sleep disorder (eTable 7 in Supplement 1).

## Discussion

In this large-scale prospective cohort study, we found that diagnosis of a psychiatric disorder was associated with an increased risk of developing T2D in young adults. During the follow-up period of 7.59 years, diagnosis of a psychiatric disorder in young adults (20-39 years) was significantly associated with an increased risk of developing T2D, regardless of sociodemographic characteristics. Young adults with schizophrenia and bipolar disorder had the highest risk of developing T2D.

Epidemiological studies^21^ have shown that psychiatric disorders are associated with an increased risk of developing T2D. The mechanisms underlying the association of T2D incidence with psychiatric disorders are multifactorial and include genetic, lifestyle, and disease-specific factors (eg, antipsychotic medication use).^22,23^ Because unhealthy lifestyle factors are more common in young adults,^24^ young adults with psychiatric disorders could have a higher risk of developing T2D. Therefore, lifestyle interventions for young adults with psychiatric disorders may be necessary to reduce the development of T2D. However, the management of young-onset T2D in young adults with psychiatric disorders is challenging and has age-specific concerns. Prospective longitudinal data should be accumulated to establish evidence-based strategies for the early prevention and detection of T2D in younger adults with psychiatric disorders. Overall, we observed that diagnosis of a psychiatric disorder was associated with an increased risk of developing T2D during a longitudinal follow-up of young adults with different types of psychiatric disorders. More research is needed to understand potential mediating mechanisms and elucidate the age-specific differences in the onset of T2D.

Although longitudinal studies have investigated the associations of different psychiatric disorders with developing T2D, they have focused on 1 specific psychiatric disorder or had small sample sizes. The current study evaluated the epidemiological association of T2D incidence with a broad range of psychiatric disorders in young adults. Our findings are consistent with those of several studies reporting that patients with schizophrenia^25^ and bipolar disorder^26^ are at least twice as likely as patients without such disorders to develop T2D. The association of clinically recognized depression,^27^ anxiety disorders,^28^ and sleep disorder^29^ with T2D incidence has been reported. Anxiety disorders are the most prevalent type of psychiatric disorder and have high comorbidity rates with other psychiatric disorders.^30^ In the current study, the association of T2D with anxiety disorder was statistically significant in the adjusted model, even though the incident rate of T2D among patients with anxiety was lower than those of the other disorders; however, after propensity score matching, the association became insignificant. We acknowledge the heterogeneity within each type of psychiatric disorder and expect that potential mediating mechanisms may explain the differences in T2D incidence.

In the exploratory subgroup analyses, we evaluated the association of T2D incidence with psychiatric disorders in young adults according to age and sex. Regarding age, the risk of developing T2D in young adults with depressive disorder, anxiety disorder, and sleep disorder was higher in the 20 to 29 year age group than in the 30 to 39 year age group. The age at the time of diagnosis of schizophrenia and bipolar disorder was not associated with a risk of developing T2D risk. A recent study^31^ indicated that early-onset schizophrenia might be associated with increased risk of developing T2D. A significant association of the diagnosis of bipolar disorder with T2D incidence has also been reported.^32^ Further research is required to confirm these findings. In our study, sex-based differences among patients with specific psychiatric disorders were statistically significant; women had a higher risk of T2D across all 5 psychiatric disorders than men. Sex-based differences among patients with specific psychiatric disorders have been widely recognized,^33^ and the results showing sex-based differences require cautious interpretation. Moreover, precisely understanding the underlying cause and mechanisms of sex-based differences is important for preventative care and to provide appropriate interventions. Although we did not investigate the underlying mechanisms of sex-specific differences in T2D incidence, our results suggest that sex should be considered as a potential effect size modifier.

Some studies^34^ have indicated an association of antipsychotic medication use with development of hyperglycemia. Use of specific antipsychotic medications in young adults has also been associated with an increased risk of developing T2D with a cumulative dose.^35^ However, the current study identified an association without considering the use of antipsychotic medication. We defined psychiatric disorders according to medical records, which could not differentiate the severity and duration of the psychiatric disorder. Moreover, since the NHIS database does not contain family histories of psychiatric disorders, genetic predisposition, and health behaviors, we were unable to consider the effect sizes of these variables. Further research is needed regarding the dosage and duration of antipsychotic medication, the time since diagnosis of a psychiatric disorder, hospitalization, and defining cases of psychiatric disorders using more sophisticated methods.

### Limitations

This longitudinal, population-based study used nationally representative data from the entire Korean population. Our study included a sufficient number of participants and had a high follow-up rate. However, this study had some limitations. First, the health examination information may pose a selection bias. We investigated patients whose psychiatric disorders were diagnosed and treated in hospitals and clinics. Patients who were not eligible for national health insurance were excluded from this study. Second, our observational study could not completely rule out residual confounding factors. Although we adjusted for various potential confounders, we could not account for reverse causality or effects of unmeasured confounders. Third, we analyzed psychiatric disorders as a dichotomous factor with T2D without considering the onset or duration of the psychiatric disorders. Hence, detection biases, to which undiagnosed T2D has partially been attributed, could not be assessed. A 2014 study^36^ has suggested that the incidence of T2D may be underestimated in patients with psychiatric disorders because of low screening rates. Fourth, we did not investigate the full spectrum of coexisting psychiatric disorders, which should be addressed in future studies.

## Conclusions

The 5 psychiatric disorders of interest were associated with a significantly increased risk of developing T2D in young adults. In particular, young adults with schizophrenia and bipolar disorder were at a higher risk of developing T2D. These results have important implications for early detection and timely intervention for T2D. More knowledge on the incidence of T2D in young adults with different psychiatric disorders is required to ensure optimal preventive approaches.

## Notes

Supplement 1. **eTable 1.** Characteristics of Participants With and Without Schizophrenia **eTable 2.** Characteristics of Participants With and Without Bipolar Disorder **eTable 3.** Characteristics of Participants With and Without Depressive Disorder **eTable 4.** Characteristics of Participants With and Without Anxiety Disorder **eTable 5.** Characteristics of Participants With and Without Sleep Disorder **eTable 6.** Baseline Characteristics of the Young Adult Population With and Without Psychiatric Disorders Before and After Propensity Score Matching **eTable 7.** Hazard Ratios of Association of Type 2 Diabetes With Psychiatric Disorders After Propensity Score Matching **eFigure.** Flow Chart of Study Population

Supplement 2.
Data Sharing Statement
