# Supplementary material for: Type 2 Diabetes and Its Association With Psychiatric Disorders in Young Adults in South Korea
Source: JAMA Netw Open. 2023 Jun 30;6(6):e2319132. doi: 10.1001/jamanetworkopen.2023.19132 (PMC10314316; doi:10.1001/jamanetworkopen.2023.19132)
Supplement: Supplement 1. — eTable 1. Characteristics of Participants With and Without Schizophrenia eTable 2. Characteristics of Participants With and Without Bipolar Disorder eTable 3. Characteristics of Participants With and Without Depressive Disorder eTable 4. Characteristics of Participants With and Without Anxiety Disorder eTable 5. Characteristics of Participants With and Without Sleep Disorder eTable 6. Baseline Characteristics of the Young Adult Population With and Without Psychiatric Disorders Before and After Propensity Score Matching eTable 7. Hazard Ratios of Association of Type 2 Diabetes With Psychiatric Disorders After Propensity Score Matching eFigure. Flow Chart of Study Population [file jamanetwopen-e2319132-s001.pdf]

Supplemental Online Content

Lee MK, Lee SY, Sohn SY, Ahn J, Han K, Lee JH. Type 2 diabetes and its association with psychiatric disorders in young adults in South Korea. *JAMA Netw Open*. 2023; 6(6):e2319132. doi:10.1001/jamanetworkopen.2023.19132

eTable 1. Characteristics of Participants With and Without Schizophrenia
eTable 2. Characteristics of Participants With and Without Bipolar Disorder
eTable 3. Characteristics of Participants With and Without Depressive Disorder
eTable 4. Characteristics of Participants With and Without Anxiety Disorder
eTable 5. Characteristics of Participants With and Without Sleep Disorder
eTable 6. Baseline Characteristics of the Young Adult Population With and Without Psychiatric Disorders Before and After Propensity Score Matching
eTable 7. Hazard Ratios of Association of Type 2 Diabetes With Psychiatric Disorders After Propensity Score Matching
eFigure. Flow Chart of Study Population

This supplemental material has been provided by the authors to give readers additional information about their work.

© 2023 Lee MK et al. *JAMA Network Open.*

eTable 1. Baseline Characteristics of the Young Adult Population With and Without Schizophrenia

	Schizophrenia		*P* value
	No (n = 6,450,583)	Yes (n = 7,408)	
Bipolar disorder (%)	8815(0.14)	1996(26.94)	<.0001
Depressive disorder (%)	177787(2.76)	3443(46.48)	<.0001
Anxiety disorder (%)	405767(6.29)	2648(35.75)	<.0001
Sleep disorder (%)	168001(2.6)	2038(27.51)	<.0001
Age (years)	30.8±4.98	31.32±5.1	<.0001
Male (%)	3817576(59.18)	4282(57.8)	0.0158
Female (%)	2633007(40.82)	3,126(42.2)	<.0001
Low income (%)	1014694(15.73)	2378(32.1)	<.0001
Heavy alcohol drinker (%)	561977(8.71)	356(4.81)	<.0001
Current smoker (%)	2231711(34.6)	2435(32.87)	0.0018
Regular physical activity (%)	829648(12.86)	1131(15.27)	<.0001
Body mass index (kg/m²)	22.95±3.57	23.72±3.99	<.0001
Systolic blood pressure (mmHg)	117.58±13.1	116.4±13.2	<.0001
Diastolic blood pressure (mmHg)	73.68±9.4	73.04±10	<.0001
Fasting plasma glucose (mg/dL)	89.49±10.61	90.25±11	<.0001
Total cholesterol (mg/dL)	184.42±35.92	185.02±35.73	0.1506
Hypertension (%)	453312(7.03)	555(7.49)	0.1181
Dyslipidemia (%)	422954(6.56)	627(8.46)	<.0001
Metabolic syndrome (%)	636139(9.86)	1054(14.23)	<.0001
Follow-up duration (years)*	7.59 (6.47-8.23)	7.24 (6.03-8.19)	<.0001

© 2023 Lee MK et al. *JAMA Network Open.*

eTable 2. Baseline Characteristics of the Young Adult Population With and Without Bipolar Disorder

	Bipolar disorder		*P* value
	No (n = 6,447,180)	Yes (n = 10,811)	
Schizophrenia (%)	5412(0.08)	1996(18.46)	<.0001
Depressive disorder (%)	176236(2.73)	4994(46.19)	<.0001
Anxiety disorder (%)	404506(6.27)	3909(36.16)	<.0001
Sleep disorder (%)	166955(2.59)	3084(28.53)	<.0001
Age (years)	30.8±4.98	31.1±5.04	<.0001
Male (%)	3816833(59.2)	5025(46.48)	<.0001
Female (%)	2630347(40.8)	5786(53.52)	<.0001
Low income (%)	1014214(15.73)	2858(26.44)	<.0001
Heavy alcohol drinker (%)	561652(8.71)	681(6.3)	<.0001
Current smoker (%)	2230693(34.6)	3453(31.94)	<.0001
Regular physical activity (%)	829161(12.86)	1618(14.97)	<.0001
Body mass index (kg/m²)	22.95±3.57	23.26±3.9	<.0001
Systolic blood pressure (mmHg)	117.58±13.1	115.52±13.31	<.0001
Diastolic blood pressure (mmHg)	73.68±9.4	72.53±9.48	<.0001
Fasting plasma glucose (mg/dL)	89.49±10.61	89.51±10.75	0.8409
Total cholesterol (mg/dL)	184.42±35.91	184.14±39.9	0.4064
Hypertension (%)	453135(7.03)	732(6.77)	0.2952
Dyslipidemia (%)	422702(6.56)	879(8.13)	<.0001
Metabolic syndrome (%)	635926(9.86)	1267(11.72)	<.0001
Follow-up duration (years)*	7.6 (6.47-8.23)	7.11 (6-8.11)	<.0001

© 2023 Lee MK et al. *JAMA Network Open.*

eTable 3. Baseline Characteristics of the Young Adult Population With and Without Depressive Disorder

	Depressive disorder		*P* value
	No (n=6,276,761)	Yes (n=181,230)	
Schizophrenia (%)	3965(0.06)	3443(1.9)	<.0001
Bipolar disorder (%)	5817(0.09)	4994(2.76)	<.0001
Anxiety disorder (%)	358118(5.71)	50297(27.75)	<.0001
Sleep disorder (%)	141740(2.26)	282999(15.61)	<.0001
Age (years)	30.79±4.98	31.42±5	<.0001
Male (%)	3742930(59.63)	78928(43.55)	<.0001
Female (%)	2533831(40.37)	102302(56.45)	<.0001
Low income (%)	983120(15.66)	33952(18.73)	<.0001
Heavy alcohol drinker (%)	548610(8.74)	13723(7.57)	<.0001
Current smoker (%)	2183140(34.78)	51006(28.14)	<.0001
Regular physical activity (%)	806762(12.85)	24017(13.25)	<.0001
Body mass index (kg/m^2)	22.96±3.57	22.58±3.63	<.0001
Systolic blood pressure (mmHg)	117.64±13.1	115.53±13.01	<.0001
Diastolic blood pressure (mmHg)	73.71±9.4	72.55±9.39	<.0001
Fasting plasma glucose (mg/dL)	89.5±10.62	89.14±10.51	<.0001
Total cholesterol (mg/dL)	184.45±35.94	183.51±35.02	<.0001
Hypertension (%)	441513(7.03)	12354(6.82)	0.0004
Dyslipidemia (%)	411063(6.55)	12518(6.91)	<.0001
Metabolic syndrome (%)	620718(9.89)	16475(9.09)	<.0001
Follow-up duration (years)*	7.6 (6.48-8.23)	7.4 (6.23-8.19)	<.0001

© 2023 Lee MK et al. *JAMA Network Open.*

eTable 4. Baseline Characteristics of the Young Adult Population With and Without Anxiety Disorder

	Anxiety disorder		*P* value
	No (n=6,049,576)	Yes (n=408,415)	
Schizophrenia (%)	4760(0.08)	2648(0.65)	<.0001
Bipolar disorder (%)	6902(0.11)	3909(0.96)	<.0001
Depressive disorder (%)	130933(2.16)	50297(12.32)	<.0001
Sleep disorder (%)	129529(2.14)	40510(9.92)	<.0001
Age (years)	30.77±4.98	31.32±5.02	<.0001
Male (%)	3632333(60.04)	189525(46.41)	<.0001
Female (%)	2417243(39.96)	218890(53.59)	<.0001
Low income (%)	944099(15.61)	72973(17.87)	<.0001
Heavy alcohol drinker (%)	531834(8.79)	30499(7.47)	<.0001
Current smoker (%)	2121131(35.06)	113015(27.67)	<.0001
Regular physical activity (%)	778415(12.87)	52364(12.82)	0.3955
Body mass index (kg/m²)	22.97±3.57	22.58±3.56	<.0001
Systolic blood pressure (mmHg)	117.68±13.1	116.05±13	<.0001
Diastolic blood pressure (mmHg)	73.74±9.4	72.82±9.33	<.0001
Fasting plasma glucose (mg/dL)	89.51±10.62	89.22±10.56	<.0001
Total cholesterol (mg/dL)	184.48±35.93	183.61±35.73	<.0001
Hypertension (%)	425467(7.03)	28400(6.95)	0.055
Dyslipidemia (%)	395815(6.54)	27766(6.8)	<.0001
Metabolic syndrome (%)	599795(9.91)	37398(9.16)	<.0001
Follow-up duration (years)*	7.6 (6.48-8.23)	7.5 (6.33-8.21)	<.0001

© 2023 Lee MK et al. *JAMA Network Open.*

eTable 5. Baseline Characteristics of the Young Adult Population With and Without Sleep Disorder

	Sleep disorder		*P* value
	No (n = 6,287,952)	Yes (n = 170,039)	
Schizophrenia (%)	5370(0.09)	2038(1.2)	<.0001
Bipolar disorder (%)	7727(0.12)	3084(1.81)	<.0001
Depressive disorder (%)	152931(2.43)	28299(16.64)	<.0001
Anxiety disorder (%)	367905(5.85)	40510(23.82)	<.0001
Age (years)	30.78±4.99	31.62±4.9	<.0001
Male (%)	3749150(59.62)	72708(42.76)	<.0001
Female (%)	2538802(40.38)	97331(57.24)	<.0001
Low income (%)	983701(15.64)	33371(19.63)	<.0001
Heavy alcohol drinker (%)	548680(8.73)	13653(8.03)	<.0001
Current smoker (%)	2185015(34.75)	49131(28.89)	<.0001
Regular physical activity (%)	807566(12.84)	23213(13.65)	<.0001
Body mass index (kg/m^2)	22.96±3.57	22.61±3.63	<.0001
Systolic blood pressure (mmHg)	117.62±13.1	115.89±12.98	<.0001
Diastolic blood pressure (mmHg)	73.7±9.4	72.86±9.38	<.0001
Fasting plasma glucose (mg/dL)	89.5±10.61	89.3±10.69	<.0001
Total cholesterol (mg/dL)	184.43±35.89	184.12±37.07	0.0004
Hypertension (%)	441941(7.03)	11926(7.01)	0.8151
Dyslipidemia (%)	411468(6.54)	12113(7.12)	<.0001
Metabolic syndrome (%)	621040(9.88)	16153(9.5)	<.0001
Follow-up duration (years)*	7.6 (6.48-8.23)	7.44 (6.24-8.19)	<.0001

© 2023 Lee MK et al. *JAMA Network Open.*

eTable 6. Baseline Characteristics of the Young Adult Population With and Without Psychiatric Disorders Before and After Propensity Score Matching

	Before Propensity Score Matching			After Propensity Score Matching		
	Psychiatric disorders		ASMD	Psychiatric disorders		ASMD
	No (n = 5,799,561)	Yes (n = 658,430)		No (n = 3,292,100)	Yes (n = 658,420)	
Age (years)	30.74±4.98	31.36±4.99	0.1237	31.32±5.01	31.36±4.99	0.0084
Male (%)	3521341(60.72)	300517(45.64)	0.3056	1498021(45.5)	300517(45.64)	0.0028
Female (%)	2278220(39.28)	357913(54.36)		1794079(54.5)	357903(54.36)	
Low income (%)	897429(15.47)	119643(18.17)	0.0721	587451(17.84)	119635(18.17)	0.0085
Heavy alcohol drinker (%)	512236(8.83)	50097(7.61)	0.0446	249607(7.58)	50097(7.61)	0.0010
Current smoker (%)	2048812(35.33)	185334(28.15)	0.1547	922694(28.03)	185333(28.15)	0.0027
Regular physical activity (%)	744991(12.85)	85788(13.03)	0.0055	424337(12.89)	85784(13.03)	0.0041
Body mass index (kg/m²)	22.99±3.56	22.61±3.59	0.1046	22.6±3.54	22.61±3.59	0.0024
Systolic blood pressure (mmHg)	117.76±13.1	115.96±13	0.1376	115.95±13.24	115.96±13	0.0008
Diastolic blood pressure (mmHg)	73.78±9.4	72.79±9.35	0.1061	72.78±9.54	72.79±9.35	0.0012
Fasting plasma glucose (mg/dL)	89.52±10.62	89.21±10.57	0.0293	89.2±10.53	89.21±10.57	0.0009
Total cholesterol (mg/dL)	184.51±35.94	183.66±35.74	0.0235	183.65±35.2	183.66±35.74	0.0003
Hypertension (%)	408830(7.05)	45037(6.84)	0.0082	222597(6.76)	45027(6.84)	0.0031
Dyslipidemia (%)	378765(6.53)	44816(6.81)	0.0111	203045(6.17)	44814(6.81)	0.0259
Metabolic syndrome (%)	576555(9.94)	60638(9.21)	0.0249	301612(9.16)	60628(9.21)	0.0016

© 2023 Lee MK et al. *JAMA Network Open.*

eTable 7 - Hazard Ratios of Association of Type 2 Diabetes With Psychiatric Disorders After Propensity Score Matching

		Number	Cases, n	Follow-up duration (person-years)	Incidence rate (per 1,000 person-years)	HR (95%CI)
Psychiatric disorder	No	3292100	67470	24052702.96	2.805	1(Ref.)
	Yes	658420	13730	4755092.97	2.887	1.038(1.019,1.057)
Schizophrenia	No	3943112	80887	28756072.81	2.813	1(Ref.)
	Yes	7408	313	51723.13	6.051	2.203(1.972,2.462)
Bipolar disorder	No	3939710	80827	28733307.87	2.813	1(Ref.)
	Yes	10810	373	74488.06	5.008	1.844(1.666,2.042)
Depressive disorder	No	3769293	77301	27508254.06	2.810	1(Ref.)
	Yes	181227	3899	1299541.87	3.000	1.08(1.046,1.115)
Anxiety disorder	No	3542112	73001	25850974.54	2.824	1(Ref.)
	Yes	408408	8199	2956821.39	2.773	0.987(0.965,1.01)
Sleep disorder	No	3780485	77255	27586799.75	2.800	1(Ref.)
	Yes	170035	3945	1220996.18	3.231	1.166(1.129,1.204)

eFigure. Flow Chart of Study Population

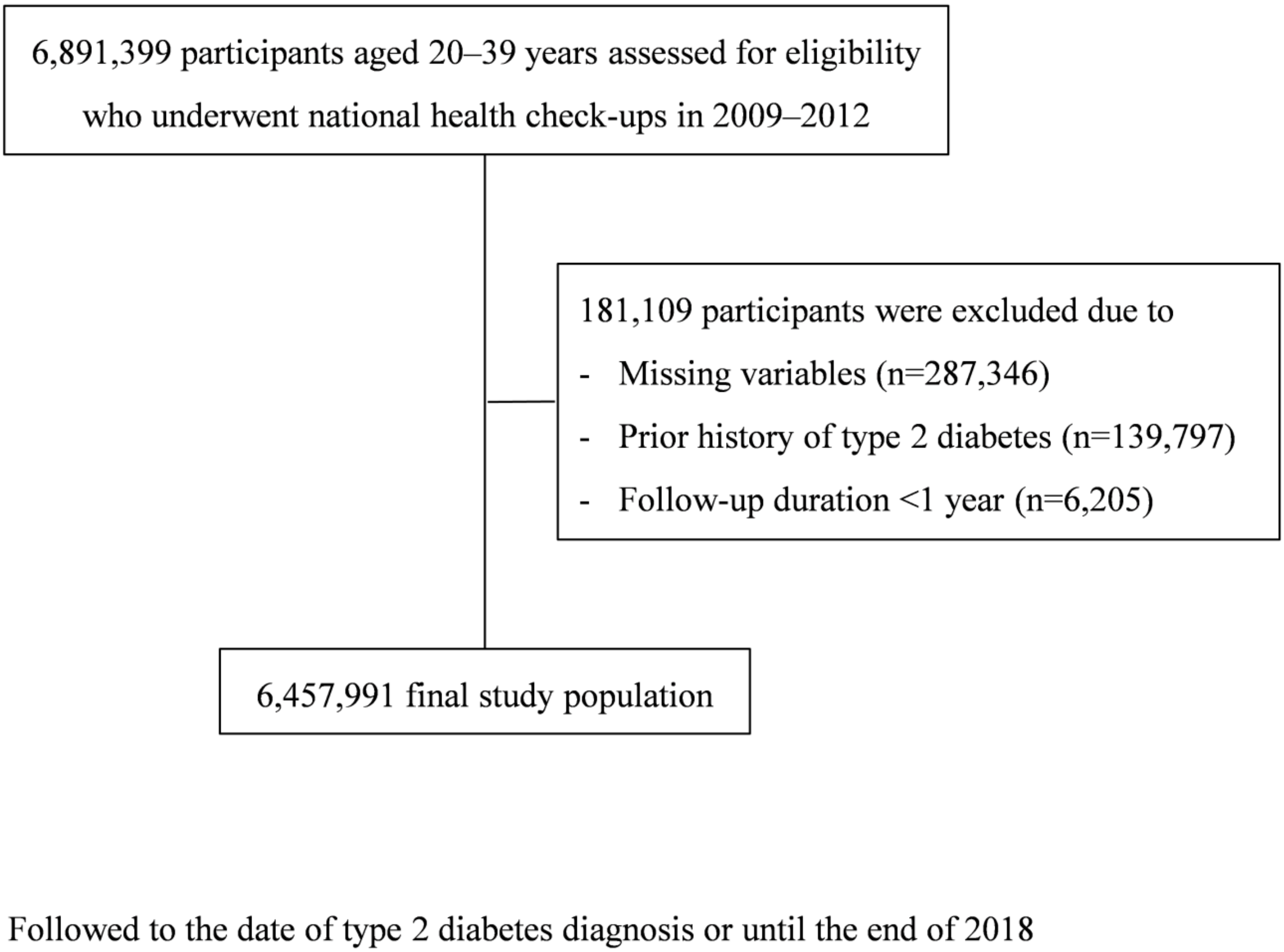

© 2023 Lee MK et al. *JAMA Network Open.*
