# Supplementary material for: Type 2 Diabetes and Its Association With Psychiatric Disorders in Young Adults in South Korea
Source: JAMA Netw Open. 2023 Jun 30;6(6):e2319132. doi: 10.1001/jamanetworkopen.2023.19132 (PMC10314316; doi:10.1001/jamanetworkopen.2023.19132)
Supplement: Supplement 2. — Data Sharing Statement [file jamanetwopen-e2319132-s002.pdf]

Data Sharing Statement

Lee. Type 2 Diabetes and Its Association With Psychiatric Disorders in Young Adults in South Korea. *JAMA Netw Open*. Published June 30, 2023.
doi:10.1001/jamanetworkopen.2023.19132

Data
Data available: Yes
Data types: Other (please specify)
Additional Information: The authors are restricted from sharing the data underlying this study because The Korean National Health Insurance Service (NHIS) owns the data. Researchers can request access on the NHIS website (https://nhiss.nhis.or.kr).
How to access data: http://nhiss.nhis.or.kr/bd/ab/bdaba000eng.do
When available: With publication

Supporting Documents
Document types: None

Additional Information
Who can access the data: None
Types of analyses: None
Mechanisms of data availability: None
Any additional restrictions: None
